# The Association of the Distance to the Hospital, Hospital Reputation, and Hospitalization Outcomes Among Patients with Stroke in China

**DOI:** 10.3390/healthcare13111276

**Published:** 2025-05-28

**Authors:** Zhenhua Qin, Yi Zhu, Jiachi Zhang, Honghong Feng, Esthefany Xu Zheng, Xiaodi Zhu, Yixiang Huang

**Affiliations:** Department of Health Policy & Management, School of Public Health, Sun Yat-sen University, No.74, Zhongshan Road 2, Guangzhou 510080, China; qinzhh5@mail2.sysu.edu.cn (Z.Q.); zhuy276@mail2.sysu.edu.cn (Y.Z.); zhangjch59@mail2.sysu.edu.cn (J.Z.); fenghh6@mail2.sysu.edu.cn (H.F.); xuzheng8@mail2.sysu.edu.cn (E.X.Z.); zhuxd9@mail2.sysu.edu.cn (X.Z.)

**Keywords:** distance to hospital, hospital reputation, hospitalization expenses, length of stay, in-hospital mortality, stroke

## Abstract

**Background**: Both distance to the hospital and hospital reputation influence patient choice of hospital; but the combined effect of these factors and how they relate to hospitalization outcomes has yet to be determined. The purpose of this study was to assess the combined influence of distance to hospital and hospital reputation on the hospitalization outcomes in patients with stroke. **Methods**: This retrospective observational study utilized data from 69,107 stroke patients hospitalized in southern Chinese megacity between 2019 and 2021. A generalized linear model was used to assess the association between hospital reputation, distance to the hospital, hospitalization costs, and the length of stay. Multivariate logistic regression was used to estimate the combined effect on in-hospital mortality. **Results**: Compared with patients who chose hospitals without a good reputation and close to home, those who chose hospitals with a good reputation had lower hospitalization costs (−0.05; 95% CI: −0.08 to −0.02), a shorter length of stay (−0.18; 95% CI: −0.20 to −0.16), and lower in-hospital mortality (0.52; 95% CI: 0.40 to 0.67). However, patients who chose hospitals with a good reputation but farther distance experienced higher hospitalization costs (0.20; 95% CI: 0.17 to 0.23). **Conclusions**: A shorter distance to the hospital and a higher reputation of the hospital are associated with lower costs and better outcomes. Our study indicates that improving outcomes for patients with stroke requires equitable distribution of quality medical resources.

## 1. Introduction

Stroke, a leading cause of death and long-term disability, imposes a significant burden on individuals, families, and healthcare systems worldwide. Globally, stroke is the second leading cause of death, with a significant increase in the prevalence and incidence of stroke among individuals under 70 years old from 1990 to 2019 [1,2]. The majority of the stroke burden is concentrated in low-income and middle-income countries, and the growth rate of the stroke burden in these countries exceeds that of high-income countries [2]. According to the report “Practical Solutions for Reducing the Global Burden of Stroke”, the global stroke burden is expected to continue to grow. By 2050, the number of people who die from stroke will increase by 50% and the associated economic losses could reach USD 23 trillion [3]. In China, stroke is the leading cause of death and disability among adults, and the disability-adjusted life years of stroke far exceed those of developed countries such as the UK, the USA, and Japan [4,5]. Investigating the reasons and major contributing factors for China’s high burden of stroke will be crucial for controlling the high prevalence trend and adverse consequences of stroke.

Stroke is a medical emergency requiring timely treatment [6,7,8], which imposes stringent demands on the geographic accessibility of healthcare resources. Existing evidence has shown that the longer distance from home to hospital is associated with delayed treatment, like thrombolytic therapy, leading to poorer health outcomes and increased economic burden [9,10,11]. Meanwhile, previous studies have observed that patients with stroke actively choose to be admitted to hospitals with higher reputation rankings [12]. The perception that a hospital with a good reputation is linked to higher quality care may result in patients prioritizing reputation over distance in the choice of hospital for stroke care. However, such hospitals are predominantly concentrated in central urban areas of major cities, forcing patients in remote regions to bear the costs of long-distance care-seeking to access high-quality services. This “quality–distance paradox” suggests that the interaction between hospital reputation and distance may impact healthcare outcomes through complex mechanisms.

While both distance to hospital and hospital reputation have been recognized to influence patient preferences [13,14], the synergistic impact of these dual factors on hospitalization outcomes remains underexplored in the existing literature [15,16,17]. While previous studies employed gravity models to analyze patient selection and evaluate the spatial accessibility of healthcare resources [18,19], our research pioneers a paradigm shift by examining downstream consequences following hospital choice. The aim of this study is to assess the combined influence of distance to hospital and hospital reputation on hospitalization outcomes for patients with stroke, including hospitalization expenses, length of stay (LoS), and in-hospital mortality. These indicators are not only outcomes of hospitalization that patients are concerned about but also reflect the efficiency and quality of medical services.

Advanced stroke centers are predominantly located in central urban areas of megacities, while residents in suburban and rural regions typically face longer travel distances to access high-reputation hospitals. This spatial inequity aligns with China’s ongoing efforts to optimize hierarchical medical systems and strengthen regional healthcare networks. Our study elucidates the combined impact of distance and hospital reputation on hospitalization outcomes, providing robust evidence to inform the spatial reallocation of high-quality stroke care resources, particularly for promoting the strategic placement of stroke care units in suburban and rural areas.

## 2. Methods

### 2.1. Data Source and Study Population

Individual-level data for hospitalized stroke patients were extracted from the electronic medical record database of a southern Chinese megacity with a population exceeding 18 million, covering the period from 1 January 2018 to 31 December 2021. The database was standardized by the National Healthcare Security Administration of China, which persistently and systematically gathers information from medical institutions. Prior to the research team’s access, patients’ unique identification numbers (IDs) were anonymized to pseudo IDs, allowing for the identification of individual patients without compromising privacy. The dataset included patient demographics such as age, gender, and residential address, as well as hospital characteristics, discharge diagnoses (both primary and secondary), and hospitalization costs. The International Classification of Diseases, Tenth Revision codes (ICD-10) were used to define stroke (ICD-10 codes: I60, I61, I62).

**Figure 1:**
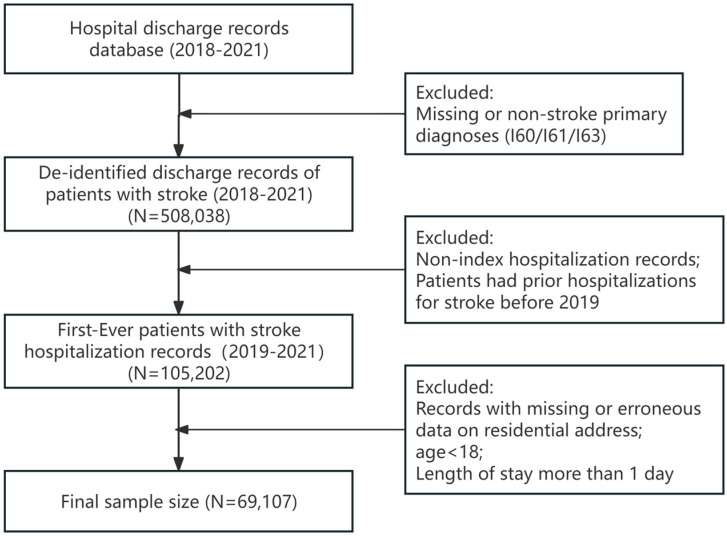
Sample selection steps.

The analysis included patients with a principle discharge diagnosis of stroke and being hospitalized between 2019 and 2021. These discharges involved patients (i) aged 18 years or older; (ii) with no prior hospitalizations for stroke before 2019; (iii) with information on residential address. A total of 69,107 patients were finally included in the study who received stroke treatment at 119 hospitals. Figure 1 illustrates the derivation of the sample.

This study was approved by the Ethics Committee of the School of Public Health, Sun Yat-Sen University (No.: 2024-38, approved on 4 March 2024).

### 2.2. Distance to Hospital and Hospital Reputation

The distance to the hospital was measured as the linear distance between the patient’s residence and the hospital attended. Both addresses were converted into longitude and latitude coordinates, and then the distance was calculated using the Haversine formula [20,21]. Both the distance to the hospital and travel time serve as a practical and standardized proxy for healthcare accessibility that has been widely validated in health services research [11,21]. While travel time may be affected by traffic patterns or road networks, straight-line distance provides a consistent, time-invariant measure that avoids the substantial data requirements and regional variability of transportation network modeling. For analysis, distances were evenly divided into three groups based on percentiles: short, medium, and long in this study.

Hospital reputation, assessed from the patient’s viewpoint, acts as a metric for evaluating hospital services [14,22]. The Fudan University Hospital Rankings are among China’s most influential hospital ranking systems, guiding patient hospital choices, informing hospital management, and shaping medical discipline development [23,24]. This ranking project is a non-profit initiative conducted by an independent third-party academic institution, primarily modeled after the specialty reputation evaluation methodology used in U.S. News & World Report’s Best Hospitals rankings. The composite score consists of two weighted components: hospital reputation score (80%) and sustainable development capability score (20%). The reputation score is derived from anonymous surveys completed by experts from the Chinese Medical Association and provincial medical associations across China who rank hospitals in their specialty; these rankings are then calculated and standardized to produce professional reputation scores. The sustainable development component evaluates objective research metrics, including SCI publications, journal impact factors, and national science and technology awards reflecting institutional research capacity. Since 2015, to better guide regional healthcare utilization, the institute has expanded its ranking system to include specialty and hospital rankings for seven major geographic regions across China. The regional rankings more accurately reflect local high-quality healthcare resource distributions. In South China, the top-ranked hospitals in both comprehensive and specialty lists are predominantly provincial tertiary hospitals and university-affiliated medical centers; their reputation scores in stroke-relevant specialties have remained relatively stable over multiple years. Considering the lagged impact of hospital rankings on patients’ medical choices, we used the 2018 rankings for the “South China Hospital Specialized Reputation Rankings” to ascertain the reputation status of hospitals. Based on whether hospitals were listed or nominated in the 2018 South China Hospital Specialty Reputation Rankings, we defined hospital reputation as a binary variable: good reputation (ranked) versus no reputation (unranked).

The independent variable was constructed as a composite measure by cross-classifying hospitals according to both the distance to the hospital (short, medium, and long distance groups) and hospital reputation (high reputation versus no reputation), resulting in six mutually exclusive categories: (1) no reputation with short distance, (2) no reputation with medium distance, (3) no reputation with long distance, (4) good reputation with short distance, (5) good reputation with medium distance, and (6) good reputation with long distance.

### 2.3. Covariates

Control variables included age (18–64, 65 years or older), gender (male or female), type of insurance, hospital level, residential area (central urban area, inner suburbs, and outer suburbs), stoke type (ischemic stroke, intracerebral hemorrhage, subarachnoid hemorrhage), and Charlson Comorbidity Index. The Charlson Comorbidity Index (CCI) is widely used to estimate the overall burden of disease in patients [25,26]. It is weighted based on the presence of 19 comorbid conditions, with varying weights assigned to different comorbidities [27,28] (Supplementary Table S1). In this study, patients’ comorbid conditions were identified from all secondary diagnoses in the database using ICD-10 coding, and the final CCI scores were categorized into three groups: 0, 1, and 2 or more. Type of insurance refers to the medical insurance plan that patients are enrolled in. In this study, insurance types contained two types: The Urban Employee’s Basic Medical Insurance (UEBMI) and the Urban–Rural Resident Basic Medical Insurance Scheme (URRBMI). The UEBMI and URRBMI are two fundamental social health insurance in China, differing in terms of the covered population and reimbursement ratios, among other aspects [29]. Previous studies have demonstrated an association between different health insurance schemes and healthcare costs as well as adverse clinical outcomes in stroke [30]. Hospital level, an essential metric for evaluating medical service quality in China, is categorized by the Chinese health administrative department into three tiers (primary, secondary, and tertiary). This classification is based on factors such as service capacity, technical expertise, and quality of care, with higher levels indicating a higher standard of treatment.

### 2.4. Outcomes

The primary outcome was hospitalization costs, defined as the total medical expenditures incurred during the index hospitalization, including both health insurance-covered expenses and out-of-pocket payments by patients, which were presented in Chinese Yuan (CNY). Secondary outcomes included the length of stay (LoS) and in-hospital mortality. LoS refers to the cumulative number of days from admission to discharge and is calculated by subtracting the admission date from the discharge date in the electronic medical records. The in-hospital mortality rate is defined as deaths that occur during hospitalization, and it is recorded by physicians in the “discharge status” field of the electronic medical records.

### 2.5. Statistical Analysis

Based on the distribution characteristics of the variables, we employed chi-square tests to compare the baseline characteristics of the study samples. The association between hospital distance, hospital reputation, and hospitalization expenses was analyzed using a generalized linear model with a gamma distribution and a log link after model selection (Supplementary Table S2). The coefficients along with their corresponding 95% confidence intervals (95% CIs) were presented in the multiple regression results. We systematically evaluated potential multicollinearity among all covariates using variance inflation factors (Supplementary Table S3). Tukey’s HSD test was employed for pairwise comparisons to control the cumulative probability of Type I errors. Furthermore, we conducted subgroup analyses stratified by age, gender, insurance type, hospital level, residential area, stroke type, and CCI score separately. In light of the characteristics of the response variable, a generalized linear model with a gamma distribution and a log link was used to assess the influence of both the distance to the hospital and hospital reputation on LoS, and multivariate logistic regression was used to assess the influence on in-hospital mortality. All analyses were performed using R (version 4.1.2), with confidence intervals calculated at the 95% level for reporting purposes. Statistical significance was determined by a 2-sided *p* value less than 0.05.

## 3. Results

### 3.1. Demographic Information

Among the 69,107 patients with stroke, 52.3% were male; 67.0% were aged 65 years or older. Additionally, 63.1% were enrolled in the Urban Employee’s Basic Medical Insurance (UEBMI), while 36.9% were covered by the Urban–Rural Resident Basic Medical Insurance Scheme. The majority suffered from ischemic stroke (91.4%), while fewer experienced intracerebral hemorrhage (7.3%) or subarachnoid hemorrhage (1.3%). Most patients opted for treatment at tertiary hospitals (78.5%). The proportion of participants with no comorbidities was 37.5%, while 33.8% had one, and 28.7% had two or more comorbidities (Table 1).

### 3.2. Association of Hospital Distance, Hospital Reputation, and Hospitalization Expenses

**Figure 2:**
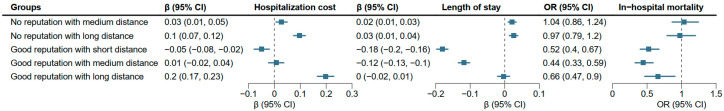
The association between hospital distance, hospital reputation, and outcomes. Figure legend: “Groups” represents the levels of the independent variable, which is a composite measure incorporating both the distance to the hospital and its reputation. A generalized linear model was employed to evaluate its association with hospitalization outcomes of patients with stroke, using no reputation with short distance as the reference group.

The generalized linear model showed differences in hospitalization expenses and length of stay among patients grouped by different combinations of hospital reputation and distance. After adjusting by age, gender, insurance type, hospital level, residential area, stroke type, and CCI score, patients in the good reputation with short distance group incurred lower hospitalization costs (−0.05, 95% CI: −0.08 to −0.02) compared to those in the group with no reputation with short distance. Conversely, patients in the no reputation with medium distance (0.03, 95% CI: 0.01 to 0.05), no reputation with long distance (0.10, 95% CI: 0.07 to 0.12), or good reputation with long distance (0.20, 95% CI: 0.17 to 0.23) faced higher medical costs(Figure 2). The results of multiple comparisons indicate that costs increased with greater distance. Notably, among all the groups examined, the group with a good reputation and long distance had the highest hospitalization costs (Supplementary Table S4).

### 3.3. Association of Hospital Distance, Hospital Reputation, and Length of Stay

After adjusting by age, gender, insurance type, hospital level, residential area, stroke type, and CCI score, patients in the no reputation with medium distance group (0.02, 95% CI: 0.01 to 0.03) and the no reputation with long distance group (0.03, 95% CI: 0.01 to 0.04) had a longer LoS compared to those in the no reputation with short distance group, although the results of multiple comparisons indicate that the differences between the two groups were not statistically significant. Patients in the good reputation with short distance group (−0.18, 95% CI: −0.20 to −0.16) and the good reputation with medium distance group (−0.12, 95% CI: −0.13 to −0.10) experienced a shorter LoS, with the good reputation and short distance group having even fewer hospital days than the good reputation with medium distance group (Figure 2).

### 3.4. Association of Hospital Distance, Hospital Reputation, and In-Hospital Mortality

After adjusting by age, gender, insurance type, hospital level, residential area, stroke type, and CCI score, there were no statistically significant differences in the risk of in-hospital mortality between the no reputation with medium distance group (OR: 1.04, 95% CI: 0.86 to 1.24) and the no reputation with long distance group (OR: 0.97, 95% CI: 0.79 to 1.20) when compared to the no reputation with short distance group. However, the risk of in-hospital case fatality was reduced for patients in the good reputation with short distance group (OR: 0.52, 95% CI: 0.40 to 0.67), the good reputation with medium distance group (OR: 0.44, 95% CI: 0.33 to 0.59), and the good reputation with long distance group (OR: 0.66, 95% CI: 0.47 to 0.90) (Figure 2).

### 3.5. Subgroup Analysis Results

The effect of a longer distance to the hospital on hospitalization costs was particularly pronounced in the subgroups living in the downtown area or inner suburbs, those with ischemic stroke, and those with a CCI score of 1 or less. Moreover, the tendency for costs to be higher at reputable hospitals compared with unreputable hospitals with increasing distance was more significant for subgroups living in non-central urban areas and for those with ischemic stroke or intracerebral hemorrhage (Table 2).

## 4. Discussion

Our study shows that among the total of 69,107 patient with stroke, a substantial number, 19,290 patients, chose to seek treatment at hospitals with a good reputation. Of these, 11,929 patients lived at a medium or long distance from these hospitals, comprising 17.2% of the total patients with stroke and 34.7% of the patients with stroke residing within medium or long distances. According to our interview results, the ambulance utilization rate among acute stroke patients in China remains relatively low, with the majority of patients arriving at hospitals through self-transportation. Among those transported by ambulance, when patients or their families explicitly request a specific hospital and formally acknowledge acceptance of all associated risks, the Emergency Medical Services system respects their preference, directing the ambulance to transfer these patients to their designated healthcare facility. In light of this, patients appeared willing to bear the added expense of traveling greater distances to access superior care provided by reputable hospitals.

Previous research has observed that longer distances to the hospital were associated with delays in seeking medical care and poorer prognosis for patients [31]. Additionally, studies have shown that hospital reputation influenced patients’ choice of destination for transfer, and transfer was associated with better patient prognosis [14,15]. Our study extends these findings by assessing the combined effect of distance to the hospital and hospital reputation on hospitalization outcomes in patients with stroke. This study reveals significant variations in hospitalization costs for stroke patients across hospitals with different distances and reputation levels. Within the same distance range, choosing high-reputation hospitals was associated with lower costs when the distance was shorter, but higher costs were observed when the distance was longer. When comparing patients selecting hospitals with similar reputation levels, increasing distance was consistently associated with higher hospitalization expenses. That is to say, patients traveling longer distances to high-reputation hospitals incur additional financial burdens. In terms of length of stay, attending a hospital farther away or without a good reputation prolonged the stay for patients with stroke. Long-distance hospital visits may lead to treatment delays and increased clinical complexity of patients’ conditions, while high-reputation hospitals typically provide superior stroke care services, which may explain these observed outcomes. The increased travel time due to longer distance could delay the time to treatment and consequently increase hospitalization costs and length of stay. Previous studies have observed that hospitals with good reputations were more likely to provide high-quality care, resulting in fewer adverse outcomes for patients [24]. As for in-hospital mortality risk, attending a hospital with a good reputation significantly reduced the in-hospital mortality for patients with stroke. This may be attributed to the provision of higher-quality medical services by hospitals with a good reputation within a short distance, which may include more advanced medical equipment, a more professional medical team, more effective treatment plans, and a reduction in unnecessary examinations and treatments. A review by Christine L Paul et al., which covered 41 related articles, indicated that shorter travel times and visiting hospitals with sufficient equipment and staffing were positively correlated with the probability of receiving thrombolytic therapy for ischemic stroke treatment [10]. Research by Jeffrey L. Saver et al. showed that early thrombolytic treatment was associated with lower mortality rates [32]. Our study aligns with these findings and expands the economic evidence. These findings underscore the importance of promoting equitable distribution of high-quality medical resources. Establishing networked regional stroke centers to provide patients with access to top-tier medical services nearby could potentially reduce treatment delays caused by distance barriers, improve clinical outcomes for stroke patients, and alleviate their financial burdens [33].

Subgroup analysis showed that the higher hospitalization costs in the group with a good reputation and long distance were more pronounced in the subgroups living in the downtown area or inner suburbs, those with ischemic stroke, and those with a CCI score not exceeding 1 compared with the reputable and short distance group, and in the subgroups residing in non-central urban areas or with ischemic stroke or intracerebral hemorrhage compared with the group without a good reputation and long distance. Regarding residential areas, traffic congestion in central urban areas can delay treatment for patients living there, potentially leading to higher hospitalization costs. Patients may be more willing to spend additional expenses if they live in the suburbs and actively choose reputable hospitals for their treatment. Treatment for cerebral infarction is time-sensitive and requires specialized equipment, making it more susceptible to missed treatment opportunities due to longer travel distances. As for the CCI score, patients with a lower CCI score may experience a more standardized stroke care process, making it easier to observe the impact of travel distance and hospital reputation on costs. However, patients with higher CCI scores may require more medical support and resources due to the presence of comorbidities [34,35], and this may mitigate the observed effects. In summary, the influence of hospital reputation and the distance from the home to the hospital on hospitalization costs is complex. These factors can be influenced by various factors such as the individual characteristics of the patient, the treatment needs, and the distribution of medical resources. It is therefore necessary to take these factors fully into account when formulating health policies in order to achieve a fairer and more effective allocation of resources, reduce the burden on patients, and improve treatment outcomes.

Our study is the first to assess the combined impact of distance to the hospital and hospital reputation on hospitalization outcomes in patients with stroke. However, there are some limitations to our study. First, the distance to hospital used in this study was the straight-line distance, which does not account for real-world conditions such as traffic congestion or road network structures, and thus cannot accurately reflect actual travel time. Nevertheless, linear distance has been widely adopted in previous studies, and the objective patient-to-hospital distance-based findings can provide clear evidence to support optimizing the equitable distribution of stroke centers. Second, The Fudan University Hospital Rankings, which we employed to represent hospital reputation, may not fully reflect patients’ trust and satisfaction with the hospital. However, this ranking has been widely used and accepted in China and can, to some extent, represent the quality of care of the hospital. Thirdly, since the data in our study originate from administrative databases, the lack of control variables such as patients’ socioeconomic status, health functional status, health literacy, and family or caregiver support may weaken the model’s efficacy. Moreover, it is impossible to track the patients’ outcomes over the long term. Nevertheless, the use of a substantial amount of objective real-world data ensures the reliability of the findings in this study. Finally, our study was conducted in a single city in China, and it may not be applicable to rural areas or areas with lower hospital density. However, the sample city serves as a representative of first-tier cities, and the results based on this city can provide valuable references for the construction and redistribution of stroke medical service systems. Subsequent research could adopt multi-source data linkages to capture modifiable factors like socioeconomic status and caregiver support availability while extending follow-up periods to investigate how hospital distance and reputation influence long-term health outcomes in stroke survivors.

## 5. Conclusions

In conclusion, this study shows that both a short distance to the hospital and a good hospital reputation significantly reduced hospitalization expenses, length of stay, and in-hospital mortality among patients with stroke. However, it was noted that the influence of hospital reputation was subject to modification by the distance to the hospital. These findings underscore the importance of balancing the equitable distribution of high-quality medical resources. Policymakers could prioritize establishing regional stroke centers in high-density suburban areas to reduce long-distance transfers. Future research could evaluate the long-term impacts of these factors while incorporating additional variables that influence patient choices and outcomes to provide a more comprehensive understanding of patient preferences for healthcare.

## Figures and Tables

**Table 1:** Demographic characteristics by distance to hospital and hospital reputation group.

	Overall (N = 69,107)	Distance to Hospital and Hospital Reputation Group						*p* Value
		No Reputation with Short Distance (N = 15,444)	No Reputation with Medium Distance (N = 16,228)	No Reputation with Long Distance (N = 18,145)	Good Reputation with Short Distance (N = 7361)	Good Reputation with Medium Distance (N = 6577)	Good Reputation with Long Distance (N = 5352)	
Age								<0.001
<65	22,795 (33.0)	4209 (27.3)	4785 (29.5)	6497 (35.8)	2236 (30.4)	2551 (38.8)	2517 (47.0)	
≥65	46,312 (67.0)	11,235 (72.7)	11,443 (70.5)	11,648 (64.2)	5125 (69.6)	4026 (61.2)	2835 (53.0)	
Gender								<0.001
Male	36,128 (52.3)	7846 (50.8)	8287 (51.1)	9997 (55.1)	3600 (48.9)	3411 (51.9)	2987 (55.8)	
Female	32,979 (47.7)	7598 (49.2)	7941 (48.9)	8148 (44.9)	3761 (51.1)	3166 (48.1)	2365 (44.2)	
Insurance type								<0.001
UEBMI	43,637 (63.1)	10,751 (69.6)	9719 (59.9)	6862 (37.8)	7003 (95.1)	5749 (87.4)	3553 (66.4)	
URRMI	25,470 (36.9)	4693 (30.4)	6509 (40.1)	11,283 (62.2)	358 (4.9)	828 (12.6)	1799 (33.6)	
Hospital level								<0.001
Primary	2027 (2.9)	1200 (7.8)	528 (3.3)	299 (1.6)	0 (0.0)	0 (0.0)	0 (0.0)	
Secondary	12,805 (18.5)	5551 (35.9)	4938 (30.4)	2316 (12.8)	0 (0.0)	0 (0.0)	0 (0.0)	
Tertiary	54,275 (78.5)	8693 (56.3)	10,762 (66.3)	15,530 (85.6)	7361 (100.0)	6577 (100.0)	5352 (100.0)	
Residential area								<0.001
Downtown area	6785 (43.9)	6323 (39.0)	2493 (13.7)	5277 (80.2)	7044 (95.7)	5277 (80.2)	1511 (28.2)	
Inner suburbs	4189 (27.1)	5618 (34.6)	5484 (30.2)	878 (13.3)	234 (3.2)	878 (13.3)	2392 (44.7)	
Outer suburbs	4470 (28.9)	4287 (26.4)	10,168 (56.0)	422 (6.4)	83 (1.1)	422 (6.4)	1449 (27.1)	
Stroke type								<0.001
Ischemic stroke	63,157 (91.4)	14,290 (92.5)	14,781 (91.1)	16,114 (88.8)	6990 (95.0)	6132 (93.2)	4850 (90.6)	
Intracerebral hemorrhage	5071 (7.3)	1017 (6.6)	1229 (7.6)	1707 (9.4)	320 (4.3)	379 (5.8)	419 (7.8)	
Subarachnoid hemorrhage	879 (1.3)	137 (0.9)	218 (1.3)	324 (1.8)	51 (0.7)	66 (1.0)	83 (1.6)	
CCI score								<0.001
0	25,909 (37.5)	5555 (36.0)	5764 (35.5)	7536 (41.5)	2522 (34.3)	2404 (36.6)	2128 (39.8)	
1	23,389 (33.8)	5077 (32.9)	5349 (33.0)	6020 (33.2)	2758 (37.5)	2346 (35.7)	1839 (34.4)	
≥2	19,809 (28.7)	4812 (31.2)	5115 (31.5)	4589 (25.3)	2081 (28.3)	1827 (27.8)	1385 (25.9)	

Abbreviations: UEBMI, The Urban Employee’s Basic Medical Insurance; URRMI, The Urban–Rural Resident Basic Medical Insurance; CCI, Charlson Comorbidity Index.

**Table 2:** Results of subgroup analysis.

	Good Reputation with Long Distance vs. Good Reputation with Short Distance		Good Reputation with Long Distance vs. No Reputation with Long Distance	
	*β* (95%CI)	*p* Value	*β* (95%CI)	*p* Value
Age				
<65	0.09 (0.02, 0.16)	0.013	0.12 (0.06, 0.17)	<0.001
≥65	0.11 (0.05, 0.17)	<0.001	0.22 (0.17, 0.27)	<0.001
Gender				
Male	0.12 (0.06, 0.18)	<0.001	0.17 (0.12, 0.22)	<0.001
Female	0.09 (0.02, 0.15)	0.008	0.18 (0.13, 0.23)	<0.001
Insurance type				
UEBMI	0.1 (0.05, 0.14)	<0.001	0.06 (0.01, 0.1)	0.014
URRMI	0.2 (0.06, 0.34)	0.006	0.33 (0.27, 0.39)	<0.001
Residential area				
Downtown area	0.08 (0.03, 0.13)	0.001	0.05 (−0.01, 0.12)	0.125
Inner suburbs	0.32 (0.19, 0.44)	<0.001	0.13 (0.08, 0.19)	<0.001
Outer suburbs	−0.03 (−0.24, 0.18)	0.775	0.3 (0.24, 0.37)	<0.001
Stroke type				
Ischemic stroke	0.1 (0.05, 0.15)	<0.001	0.17 (0.13, 0.21)	<0.001
Intracerebral hemorrhage	0.15 (−0.03, 0.33)	0.105	0.18 (0.07, 0.29)	0.001
Subarachnoid hemorrhage	0.12 (−0.22, 0.45)	0.498	0.09 (−0.11, 0.3)	0.371
CCI score				
0	0.15 (0.07, 0.23)	<0.001	0.23 (0.17, 0.28)	<0.001
1	0.11 (0.04, 0.19)	0.002	0.11 (0.04, 0.17)	0.001
≥2	0.03 (−0.06, 0.11)	0.534	0.17 (0.1, 0.24)	<0.001

Abbreviations: UEBMI, The Urban Employee’s Basic Medical Insurance; URRMI, The Urban-Rural Resident Basic Medical Insurance; CCI, Charlson Comorbidity Index.

## Data Availability

The original data cannot be publicly shared due to the inclusion of patients’ private address information. De-identified data may be made available to qualified researchers subject to strict data protection agreements and ethical review. The detailed process and conditions for requesting data access can be found at https://docs.qq.com/doc/DS1JPeUJVQ3ROR09o# (accessed on 5 September 2024).

## Supplementary Materials

The following supporting information can be downloaded at: https://www.mdpi.com/article/10.3390/healthcare13111276/s1, Table S1. ICD-10 Coding Algorithms and Index Weight for Charlson Comorbidities. Table S2. Association between Outcomes and Distance to Hospital and Hospital Reputation Group. Table S3. Multicollinearity diagnostic tests. Table S4. Results of Tukey’s HSD.
