# Supplementary material for: The Association of the Distance to the Hospital, Hospital Reputation, and Hospitalization Outcomes Among Patients with Stroke in China
Source: Healthcare (Basel). 2025 May 28;13(11):1276. doi: 10.3390/healthcare13111276 (PMC12154349; doi:10.3390/healthcare13111276)
Supplement: Supplementary file 1 [file healthcare-13-01276-s001.zip › healthcare-3581595-supplementary.pdf]

Table S1 ICD-10 Coding Algorithms and Index Weight for Charlson Comorbidities

Comorbidities	ICD-10	Index Weight
Myocardial infarction	I21.x, I22.x, I25.2	1
Congestive heart failure	I09.9, I11.0, I13.0, I13.2, I25.5, I42.0,I42.5–I42.9, I43.x, I50.x, P29.0	1
Peripheral vascular disease	I70.x, I71.x, I73.1, I73.8, I73.9, I77.1,I79.0, I79.2, K55.1, K55.8, K55.9,Z95.8, Z95.9	1
Cerebrovascular disease	G45.x, G46.x, H34.0, I60.x–I69.x	1
Dementia	F00.x–F03.x, F05.1, G30.x, G31.1	1
Chronic pulmonary disease	I27.8, I27.9, J40.x–J47.x, J60.x–J67.x,J68.4, J70.1, J70.3	1
Rheumatic disease	M05.x, M06.x, M31.5, M32.x–M34.x,M35.1, M35.3, M36.0	1
Peptic ulcer disease	K25.x–K28.x	1
Mild liver disease	B18.x, K70.0–K70.3, K70.9,K71.3–K71.5, K71.7, K73.x, K74.x,K76.0, K76.2–K76.4, K76.8, K76.9,Z94.4	1
Diabetes without chronic complication	E10.0, E10.1, E10.6, E10.8, E10.9,E11.0, E11.1, E11.6, E11.8, E11.9,E12.0, E12.1, E12.6, E12.8, E12.9,E13.0, E13.1, E13.6, E13.8, E13.9,E14.0, E14.1, E14.6, E14.8, E14.9	1
Diabetes with chronic complication	E10.2–E10.5, E10.7, E11.2–E11.5,E11.7, E12.2–E12.5, E12.7, E13.2–E13.5, E13.7, E14.2–E14.5, E14.7	2
Hemiplegia or paraplegia	G04.1, G11.4, G80.1, G80.2, G81.x,G82.x, G83.0–G83.4, G83.9	2
Renal disease	I12.0, I13.1, N03.2–N03.7, N05.2–N05.7, N18.x, N19.x, N25.0, Z49.0–Z49.2, Z94.0, Z99.2	2
Any malignancy，including lymphoma and leukemia, except malignant neoplasm of skin	C00.x–C26.x, C30.x–C34.x, C37.x–C41.x, C43.x, C45.x–C58.x, C60.x–C76.x, C81.x–C85.x, C88.x,C90.x–C97.x	2
Moderate or severe liver disease	I85.0, I85.9, I86.4, I98.2, K70.4,K71.1, K72.1, K72.9, K76.5, K76.6,K76.7	3
Metastatic solid tumor	C77.x–C80.x	6
AIDS/HIV	B20.x–B22.x, B24.x	6

TableS2 Association between Outcomes and Distance to Hospital and Hospital Reputation Group

Variables	Model1	Model2[a]	Model3[b]
Hospitalization cost [β (95%CI)]			
No reputation and short distance	Ref	Ref	Ref
No reputation and medium distance	0.11(0.08,0.13)***	0.12(0.10,0.15)***	0.03(0.01,0.05)*
No reputation and long distance	0.19(0.16,0.22)***	0.34(0.32,0.37)***	0.10(0.07,0.12)***
Good reputation and short distance	0.3(0.26,0.33)***	0.13(0.10,0.17)***	-0.05(-0.08,-0.02)**
Good reputation and medium distance	0.32(0.29,0.36)***	0.21(0.18,0.25)***	0.01(-0.02,0.04)
Good reputation and long distance	0.4(0.36,0.44)***	0.46(0.42,0.50)***	0.20(0.17,0.23)***
$R2$	0.023	0.082	0.298
AIC	1481837	1477087	1456978
Length of stay [β (95%CI)]			
No reputation and short distance	Ref	Ref	Ref
No reputation and medium distance	0.03(0.01,0.04)***	0.04(0.02,0.05)***	0.02(0.01,0.03)***
No reputation and long distance	-0.01(-0.02,0.00)*	0.07(0.05,0.08)***	0.03(0.01,0.04)***
Good reputation and short distance	-0.09(-0.1,-0.07)***	-0.17(-0.18,-0.15)***	-0.18(-0.2,-0.16)***
Good reputation and medium distance	-0.05(-0.06,-0.03)***	-0.10(-0.12,-0.08)***	-0.12(-0.13,-0.1)***
Good reputation and long distance	-0.01(-0.03,0.01)	0.03(0.01,0.04)**	0.00(-0.02,0.01)
$R2$	0.003	0.022	0.059
AIC	405105	403681	400871
In-hospital mortality [OR (95%CI)]			
No reputation and short distance	Ref	Ref	Ref
No reputation and medium distance	1.02(0.86,1.22)	1.13(0.94,1.34)	1.04(0.86,1.24)
No reputation and long distance	0.68(0.56,0.82)***	1.16(0.95,1.41)	0.97(0.79,1.20)
Good reputation and short distance	0.76(0.59,0.97)*	0.46(0.36,0.59)***	0.52(0.40,0.67)***
Good reputation and medium distance	0.57(0.43,0.76)***	0.41(0.30,0.54)***	0.44(0.33,0.59)***
Good reputation and long distance	0.53(0.39,0.73)***	0.69(0.49,0.94)*	0.66(0.47,0.90)*
$R2$	0.005	0.040	0.177
AIC	9653.1	9325.6	8003

[a] Adjusted by age, gender, insurance type, residential area

[b] Adjusted by age, gender, insurance type, hospital Level, residential area, stroke type, CCI score

* $P < 0\ 05$, ** $P < 0.01$, and *** $P < 0.001$.

Table S3 Multicollinearity diagnostic tests

Variables	GVIF	Df	Adjusted GVIF
Age	1.11	1.00	1.05
Gender	2.11	1.00	2.05
Hospital level	3.11	2.00	3.05
Insurance type	4.11	1.00	4.05
Stroke type	5.11	2.00	5.05
CCI score	6.11	2.00	6.05
Residential area	7.11	2.00	7.05
Reputation-distance group	8.11	5.00	8.05

Table S4 Results of Tukey's HSD

	β (95%CI)	
**Characteristics**	**Unadjusted**	**Adjusted**
hospitalization cost		
No reputation and medium distance vs no reputation and short distance	0.11 (0.07, 0.14)	0.03 (0, 0.06)
No reputation and long distance vs no reputation and short distance	0.19 (0.15, 0.23)	0.1 (0.06, 0.13)
Good reputation and short distance vs no reputation and short distance	0.3 (0.25, 0.34)	-0.05 (-0.09, -0.01)
Good reputation and medium distance vs no reputation and short distance	0.32 (0.27, 0.37)	0.01 (-0.04, 0.05)
Good reputation and long distance vs no reputation and short distance	0.4 (0.34, 0.45)	0.2 (0.15, 0.24)
No reputation and long distance vs no reputation and medium distance	0.08 (0.05, 0.12)	0.07 (0.04, 0.1)
Good reputation and short distance vs no reputation and medium distance	0.19 (0.14, 0.24)	-0.08 (-0.12, -0.03)
Good reputation and medium distance vs no reputation and medium distance	0.21 (0.16, 0.26)	-0.02 (-0.06, 0.02)
Good reputation and long distance vs no reputation and medium distance	0.29 (0.24, 0.35)	0.17 (0.13, 0.21)
Good reputation and short distance vs no reputation and long distance	0.11 (0.06, 0.15)	-0.15 (-0.19, -0.1)
Good reputation and medium distance vs no reputation and long distance	0.13 (0.08, 0.18)	-0.09 (-0.13, -0.05)
Good reputation and long distance vs no reputation and long distance	0.21 (0.16, 0.26)	0.1 (0.06, 0.14)
Good reputation and medium distance vs good reputation and short distance	0.03 (-0.03, 0.08)	0.06 (0.01, 0.1)
Good reputation and long distance vs good reputation and short distance	0.1 (0.04, 0.16)	0.25 (0.19, 0.3)
Good reputation and long distance vs good reputation and medium distance	0.08 (0.02, 0.14)	0.19 (0.14, 0.24)
